# Structural connectome-based predictive modeling of cognitive deficits in treated glioma patients

**DOI:** 10.1093/noajnl/vdad151

**Published:** 2023-11-15

**Authors:** Michel Friedrich, Christian P Filss, Philipp Lohmann, Felix M Mottaghy, Gabriele Stoffels, Carolin Weiss Lucas, Maximilian I Ruge, N Jon Shah, Svenja Caspers, Karl-Josef Langen, Gereon R Fink, Norbert Galldiks, Martin Kocher

**Affiliations:** Institute of Neuroscience and Medicine (INM-1, INM-3, INM-4, INM-11), Forschungszentrum Juelich, Juelich, Germany; Institute of Neuroscience and Medicine (INM-1, INM-3, INM-4, INM-11), Forschungszentrum Juelich, Juelich, Germany; Institute of Neuroscience and Medicine (INM-1, INM-3, INM-4, INM-11), Forschungszentrum Juelich, Juelich, Germany; Department of Nuclear Medicine, RWTH University Hospital Aachen, RWTH University Aachen, Aachen, Germany; Institute of Neuroscience and Medicine (INM-1, INM-3, INM-4, INM-11), Forschungszentrum Juelich, Juelich, Germany; Department of General Neurosurgery, Center for Neurosurgery, Faculty of Medicine and University Hospital Cologne, University of Cologne, Cologne, Germany; Center of Integrated Oncology (CIO), Universities of Aachen, Bonn, Cologne, and Duesseldorf, Germany; Center of Integrated Oncology (CIO), Universities of Aachen, Bonn, Cologne, and Duesseldorf, Germany; Department of Stereotaxy and Functional Neurosurgery, Center for Neurosurgery, Faculty of Medicine and University Hospital Cologne, Cologne, Germany; Institute of Neuroscience and Medicine (INM-1, INM-3, INM-4, INM-11), Forschungszentrum Juelich, Juelich, Germany; Juelich-Aachen Research Alliance (JARA), Section JARA-Brain, Juelich, Germany; Department of Neurology, RWTH University Hospital Aachen, RWTH University Aachen, Aachen, Germany; Institute of Neuroscience and Medicine (INM-1, INM-3, INM-4, INM-11), Forschungszentrum Juelich, Juelich, Germany; Institute for Anatomy I, Medical Faculty and University Hospital Duesseldorf, Heinrich Heine University Duesseldorf, Duesseldorf, Germany; Institute of Neuroscience and Medicine (INM-1, INM-3, INM-4, INM-11), Forschungszentrum Juelich, Juelich, Germany; Department of Nuclear Medicine, RWTH University Hospital Aachen, RWTH University Aachen, Aachen, Germany; Center of Integrated Oncology (CIO), Universities of Aachen, Bonn, Cologne, and Duesseldorf, Germany; Institute of Neuroscience and Medicine (INM-1, INM-3, INM-4, INM-11), Forschungszentrum Juelich, Juelich, Germany; Department of Neurology, Faculty of Medicine and University Hospital Cologne, University of Cologne, Cologne, Germany; Institute of Neuroscience and Medicine (INM-1, INM-3, INM-4, INM-11), Forschungszentrum Juelich, Juelich, Germany; Center of Integrated Oncology (CIO), Universities of Aachen, Bonn, Cologne, and Duesseldorf, Germany; Department of Neurology, Faculty of Medicine and University Hospital Cologne, University of Cologne, Cologne, Germany; Institute of Neuroscience and Medicine (INM-1, INM-3, INM-4, INM-11), Forschungszentrum Juelich, Juelich, Germany; Center of Integrated Oncology (CIO), Universities of Aachen, Bonn, Cologne, and Duesseldorf, Germany; Department of Stereotaxy and Functional Neurosurgery, Center for Neurosurgery, Faculty of Medicine and University Hospital Cologne, Cologne, Germany

**Keywords:** brain networks, cognitive functions, diffusion-weighted imaging, glioma, structural connectivity

## Abstract

**Background:**

In glioma patients, tumor growth and subsequent treatments are associated with various types of brain lesions. We hypothesized that cognitive functioning in these patients critically depends on the maintained structural connectivity of multiple brain networks.

**Methods:**

The study included 121 glioma patients (median age, 52 years; median Eastern Cooperative Oncology Group performance score 1; CNS-WHO Grade 3 or 4) after multimodal therapy. Cognitive performance was assessed by 10 tests in 5 cognitive domains at a median of 14 months after treatment initiation. Hybrid amino acid PET/MRI using the tracer O-(2-[^18^F]fluoroethyl)-L-tyrosine, a network-based cortical parcellation, and advanced tractography were used to generate whole-brain fiber count-weighted connectivity matrices. The matrices were applied to a cross-validated machine-learning model to identify predictive fiber connections (edges), critical cortical regions (nodes), and the networks underlying cognitive performance.

**Results:**

Compared to healthy controls (*n* = 121), patients’ cognitive scores were significantly lower in 9 cognitive tests. The models predicted the scores of 7/10 tests (median correlation coefficient, 0.47; range, 0.39–0.57) from 0.6% to 5.4% of the matrix entries; 84% of the predictive edges were between nodes of different networks. Critically involved cortical regions (≥10 adjacent edges) included predominantly left-sided nodes of the visual, somatomotor, dorsal/ventral attention, and default mode networks. Highly critical nodes (≥15 edges) included the default mode network’s left temporal and bilateral posterior cingulate cortex.

**Conclusions:**

These results suggest that the cognitive performance of pretreated glioma patients is strongly related to structural connectivity between multiple brain networks and depends on the integrity of known network hubs also involved in other neurological disorders.

## Graphical Abstract

**Graphical Abstract:**
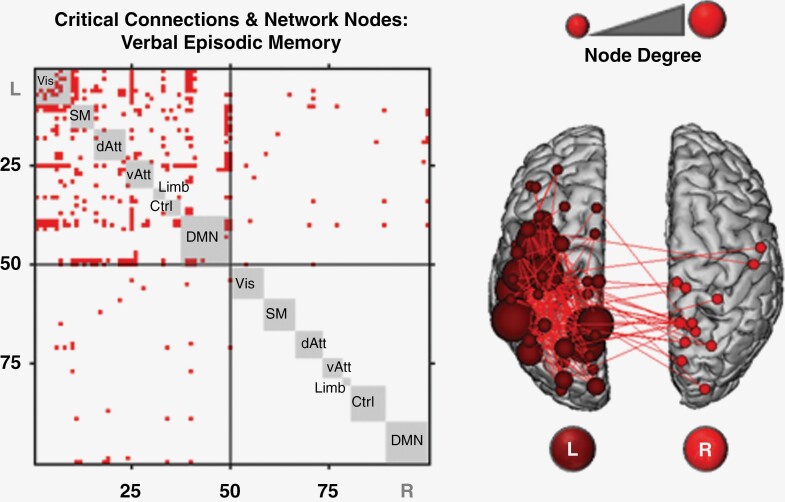

**Key Points**

- Cognitive performance is closely related to structural connectivity between multiple brain networks.
- Critically affected cortical nodes are mainly located in the left hemisphere.
- Involvement of bilateral cortical hubs known from other neurological disorders.

**Importance of the Study**

We here studied the association of whole-brain structural connectivity with cognitive performance in pretreated CNS WHO Grade 3 or 4 glioma patients through a network-based approach that included a recent cortical parcellation, advanced tractography methods, and a well-established method for connectome-based predictive modeling. We found that reduced fiber numbers in subsets of connections between different brain networks were significantly related to performance in various cognitive domains. Critical cortical regions, identified by their adjacency to predictive connections, were mainly located in the left hemisphere but also included bilateral cortical hubs, such as the precuneus and posterior cingulate cortex, which also play a significant role in other neurological diseases such as Alzheimer’s disease. This finding implies that cognitive decline in treated brain tumor patients shares a common mechanism with other psychiatric and neurological disorders.

Gliomas are the most common malignant primary brain tumors in adults.^1^ The prognosis of patients with gliomas mainly depends on molecular and histomorphologic tumor features that determine the growth rate and pattern of invasion into the normal brain tissue.^2^ Apart from a reduced survival time, glioma patients frequently experience disturbances of cognitive function^3^ that are potentially related to the structural damage imposed on the brain by the tumor itself or therapeutic interventions. Compared to other neurological disorders, such as stroke, multiple sclerosis, or dementia, the pattern of brain lesions in glioma patients is more heterogeneous, as it may comprise brain infiltration by residual or recurrent tumor growth, disruption of the blood-brain barrier, neurosurgical resection of cortical or subcortical brain tissue, or radiation- or chemotherapy-induced damage of white matter.^4^

So far, clinical research in neuro-oncology has primarily aimed at identifying selected vulnerable structures at risk of neurologic or cognitive deficits, such as the motor or language pathways (neurosurgery) or the hippocampus (radiation therapy),^5^ which in turn are preferably spared from treatment-related damage. The relationship between white matter alterations and cognitive deficits in glioma patients has been mainly established in the perioperative setting where single, anatomically defined tracts and their associated functions were identified. These include, among others, the right frontal aslant tract (executive functions, attention shift, verbal fluency),^6^ the right superior longitudinal fascicle/frontostriatal tract/orbitofrontal cortex (mentalizing/visuospatial function),^7–10^ and the right inferior frontostriatal tract/inferior frontal gyrus (inference control processes).^11^ Moreover, radiation-induced alterations in the parahippocampal cingulum of the medial temporal lobes correlated with a decline in verbal memory and verbal fluency.^12^

However, a growing body of evidence indicates that the long-term outcome of higher-order cognitive functioning in glioma patients depends on the preserved integrity of several distributed networks rather than individual nodes or tracts,^13–16^ which has led several groups to investigate the relation between cognitive outcome and structural connectivity in glioma on a more network-oriented level.^17–19^ We also took this approach here and hypothesized that the various structural brain lesions in treated glioma patients impair cognitive functions through a common mechanism, namely the reduced structural connectivity between cortical regions, resulting from altered integrity of the cortical gray matter or the adjacent white matter fiber tracts. Therefore, we constructed whole-brain structural connectomes in pretreated patients with CNS WHO Grade 3 or 4 gliomas characterized according to the 2021 WHO classification of Tumors of the CNS,^2^ and used advanced structural and diffusion-weighted MRI^20^ as well as amino acid PET^21^ imaging techniques to identify fiber tracts and structural brain lesions. In addition, a recent functional parcellation of the cortex^22^ and tractography tools capable of reasonable fiber tracking within or close to tumor- or treatment-related lesions were applied.^23,24^ The individual connectomes were used to develop a predictive machine-learning-based model^25^ that identified networks, nodes, and connecting fiber tracts critical for performance in different cognitive domains.

## Patients and Methods

### Patient Characteristics

From February, 2018 to September, 2020, we prospectively evaluated 121 pretreated glioma patients (73 men, 48 women; mean age, 51.6 ± 11.6 years) who had undergone multimodal therapy, including resection, radiotherapy, alkylating chemotherapy, or combinations thereof (Supplementary Table 1). Patients were referred for follow-up from main academic institutions that had regular access to the 3T hybrid PET/MR imaging facility where simultaneous PET/MR imaging was performed using the radiolabeled amino acid O-(2-[^18^F]fluoroethyl)-L-tyrosine (^18^F-FET) at different time points after first-line therapy (median time, 14 months; range, 1–214 months). ^18^F-FET PET is a sensitive method for early assessment of residual metabolically active tumors after surgery, evaluation of response to adjuvant chemotherapy using alkylating agents, and differentiation of tumor relapse from treatment-related changes.^26^

The inclusion criteria comprised a favorable general condition defined by a performance score of 0 or 1 according to the Eastern Cooperative Oncology Group^27^ criteria, absence of major depression, and fluency in the German language. In case of a history of seizures, appropriate anticonvulsive medication was mandatory. Patients were screened and registered for the study by phone calls, reviewed on the day of imaging, and were included in the study after providing informed written consent following the Declaration of Helsinki. The local ethics committee approved the protocol (17–365). Of the 121 patients included, 104 (86%) had completed primary treatment according to the guidelines at the time of diagnosis. As shown in Supplementary Table 1, patients had either received tumor resection (*n* = 108) or biopsy (*n* = 13), and the majority (*n* = 100) had undergone at least 1 series of local radiotherapy (60 ± 2 Gy in 92% of patients) at a median interval of 13 months (range, 2–213 months) between the start of radiotherapy and imaging. Fourteen patients had 2 radiotherapy series. In 6 patients, planned postoperative radiotherapy/chemo-radiotherapy was pending; in 11 patients, adjuvant chemotherapy was incomplete. In order to quantify treatment intensity, the number of different types of oncologic interventions was assessed and analyzed about cognitive outcome.

The study included patients with adult-type diffuse glioma of Grades 3 and 4 according to the 2021 WHO classification.^2^ All original neuropathological reports were re-classified mainly based on the isocitrate dehydrogenase (IDH)-gene mutation and 1p/19q loss-of-heterozygosity status. Most of the patients suffered from an IDH-wildtype glioblastoma (60%), but CNS WHO Grade 3 IDH-mutant astrocytomas (12%) and CNS WHO Grade 3 IDH-mutant 1p/19q co-deleted oligodendrogliomas (11%) were also prevalent. A total of 72 patients (60%) had anticonvulsive medication, and 81 patients (67%) had mild neurological (48%) or other symptoms (19%) without requiring assistance for personal needs. All patients except 1 were right-handed. Based on clinical deterioration, MRI findings, and ^18^F-FET PET results, the diagnosis of glioma relapse was obtained in 58 of 121 patients.

### Imaging Protocols

Simultaneous MR/PET imaging was performed on a 3T hybrid scanner (Siemens Trim-TRIO/BrainPET, Siemens Medical Systems, Erlangen, Germany) equipped with a PET insert. ^18^F-FET PET images were obtained as described in detail before.^28^ The presence or absence of metabolic active residual/recurrent tumor sites was assessed by a nuclear medicine physician (K.-J.L.) from the summed activity from 20 to 40 min post-injection and the time-activity curves according to established protocols.^26^

The MRI protocol comprised a 3D high-resolution T1-weighted magnetization-prepared rapid acquisition gradient echo (MPRAGE) native scan (176 slices; TR = 2250 ms; TE = 3.03 ms; field of view (FoV) = 256 × 256 mm^2^; flip angle = 9°; voxel size = 1 × 1 × 1 mm^3^), a contrast-enhanced MPRAGE scan recorded after injection of gadolinium-based contrast agent, a T2-weighted sampling perfection with application-optimized contrasts using different flip angle evolution (SPACE) scan (176 slices; repetition time TR = 3.2 ms; echo time TE = 417 ms; FoV = 256 × 256 mm^2^; voxel size = 1 × 1 × 1 mm^3^), and a T2-weighted fluid-attenuated inversion recovery (T2/FLAIR) scan (25 slices; TR = 9000 ms; TE = 3.86 ms; FoV = 220 × 220 mm^2^; flip angle = 150°; voxel size = 0.9 × 0.9 × 4 mm^3^).

High-angular-resolution diffusion imaging (HARDI) measurements were acquired using a diffusion-weighted double-echo echo-planar imaging (EPI) sequence (55 slices; TR = 8000 ms; TE = 112 ms; b-values (gradient directions) = 0 (13, interleaved) and 2700 s/mm^2^ (120); voxel size = 2.4 × 2.4 × 2.4 mm^3^). Afterward, a nondiffusion-weighted (*b* = 0) volume was acquired with the same parameters but with a reversed phase-encoding direction needed for the EPI distortion correction.

### Cognitive Performance

Cognitive performance was assessed on the day of imaging and based on 10 cognitive tests selected from a more extensive test battery developed for the 1000BRAINS study, a population-based cohort study that included over 1300 older subjects and investigated environmental and genetic influences on the interindividual variability of brain structure, function, and connectivity in the aging brain.^20^ The applied test and the respective cognitive domains are shown in Supplementary Table 2. They included tests for attention/processing speed (Trail-making Test A [TMT-A]), executive function/concept shifting (Trail-making Test B [TMT-B]), semantic word fluency (Imagined Shopping Tour) and language processing (Number Transcoding), as well as tests for verbal working memory (Digit Span forward/backward), verbal episodic memory (Word List, immediate and delayed recall) and visuospatial working memory (Corsi Block Tapping test forward/backward).

For the generation of a control group, 121 healthy subjects who had performed the same cognitive tests were selected from the 1000BRAINS study. Propensity score matching^29^ (R software package, https://www.r-project.org/) was applied to build a cohort that matched the patient population in terms of sex, age, and educational level according to the International Standard Classification of Education (ISCED) classification (http://uis.unesco.org/sites/default/files/documents/international-standard-classification-of-education-1997-en_0.pdf). This procedure resulted in a control group that broadly resembled the patient group (age 51.7 ± 11.5 vs 51.6 ± 11.6 years, 2-sided *t*-test *P* = .96; men/women 75/46 vs 73/48, 2-sided Chi-square test *P* = .90; ISCED-level 7.4 ± 1.7 vs 7.1 ± 2.1, 2-sided Mann–Whitney U-test *P* = .41). The cognitive deficits of the patients were classified as clinically relevant if their scores were lower than the mean—1.5× standard deviation of the control group.

### Whole-brain Structural Connectome

**Figure 1.**
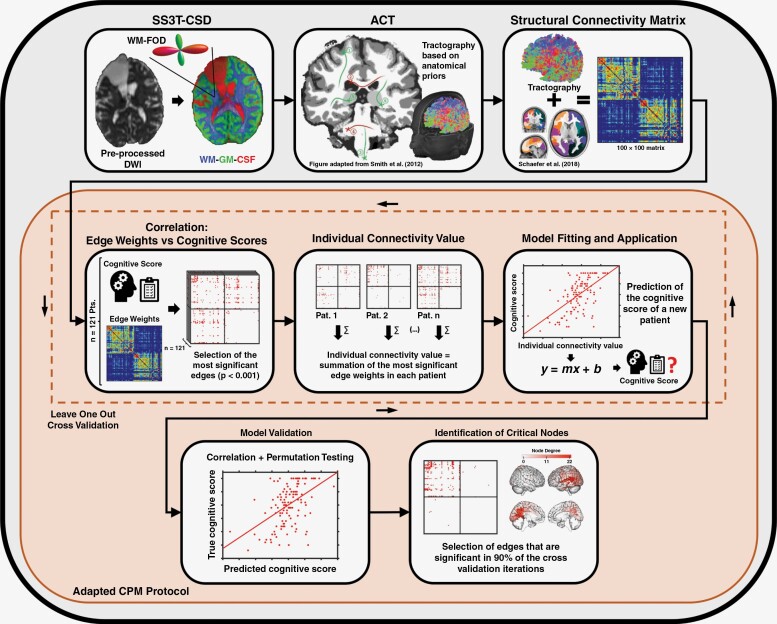
Overview of methods for determining the whole-brain structural connectome and generating and validating a connectome-based predictive model (CPM) for cognitive functioning in glioma patients following multimodality treatment. SS3T-CSD, single-shell 3-tissue constrained spherical deconvolution; WM, white matter; FOD, fiber orientation distribution function; DWI, diffusion-weighted magnetic resonance imaging; GM, gray matter; CSF, cerebrospinal fluid; ACT, Anatomically Constrained Tractography; Pts, patients; Pat, patient.

The main steps for determining the whole-brain structural connectome and prediction modeling of cognitive performance are shown in Figure 1. We used the tractography imaging pipeline based on the GitHub-fork MRtrix3Tissue (https://3tissue.github.io), a recently developed modification of the widely accepted fiber-tracking software MRtrix3 (https://www.mrtrix.org). The novel single-shell 3-tissue constrained spherical deconvolution (SS3T-CSD) method generates estimates of white matter fiber orientation distribution functions (FODs) as bias-free as possible, even within different compartments infiltrated by the tumor.^23,30,31^ This is mainly achieved by estimating the composition of each voxel in terms of white-matter-like, gray-matter-like, and cerebrospinal fluid-like tissue components, which are computed from single-shell HARDI data (single *b*-value 2700 s/mm^2^ and nondiffusion-weighted images). CSD-based fiber mapping assumes that the diffusion-weighted MRI signal results from the spherical convolution of a response function with the underlying FOD function.^32^ The response function, which is determined from the diffusion-weighted data itself, represents the expected MR signal from a pure white matter (a single-oriented white matter fiber bundle), gray matter, or cerebrospinal fluid voxel. Unlike the clinically widely used diffusion tensor model, CSD models can resolve multiple fiber orientations within an image voxel.

The HARDI data underwent image preprocessing following published recommendations (https://osf.io/ht7zv) and comprised corrections for EPI distortion, eddy current, motion distortion, and bias field. An unsupervised method was used to estimate the tissue-specific white matter, gray matter, and cerebrospinal fluid response functions from the preprocessed HARDI data. The response functions for each tissue compartment were averaged across all patients, and the tissue component fractions were corrected for the effects of residual intensity inhomogeneities by global intensity normalization^33^ to ensure that FODs estimated by SS3T-CSD^30^ were comparable within this group study. The subsequent fiber mapping was based on Anatomically Constrained Tractography, which poses physiological restrictions on the behavior of healthy neuronal fibers in terms of their propagation and termination.^24^ These assumptions were lifted in the area of pathologic tissue by masking out the entire lesioned areas. For this purpose, resection cavities were manually contoured by a radiation oncologist (M.K.), the T1-contrast-enhancing lesions and T2/FLAIR hyperintensities were automatically segmented using the deep-learning-based software HD-GLIO-AUTO (https://github.com/NeuroAI-HD/HD-GLIO-AUTO), and ^18^F-FET PET segmentation was implemented by an FSL (https://fsl.fmrib.ox.ac.uk/fsl/fslwiki) custom script using a tumor-to-brain ratio of 1.6 (20–40 min summed activity) as the lower threshold.^34^ All segmentations were visually inspected, manually corrected, and added to form a composite lesion mask. Besides the default settings of MRtrix3Tissue, the number of seed points was set to a constant of 4 million seeds randomly placed in a whole-brain mask, the backtrack option was enabled, and the cutoff value for FOD amplitude was set to 0.01. In a former study,^4^ we found that this setup could reasonably identify fibers passing through and near tumor tissue and the surrounding brain structures.

**Figure 2.**
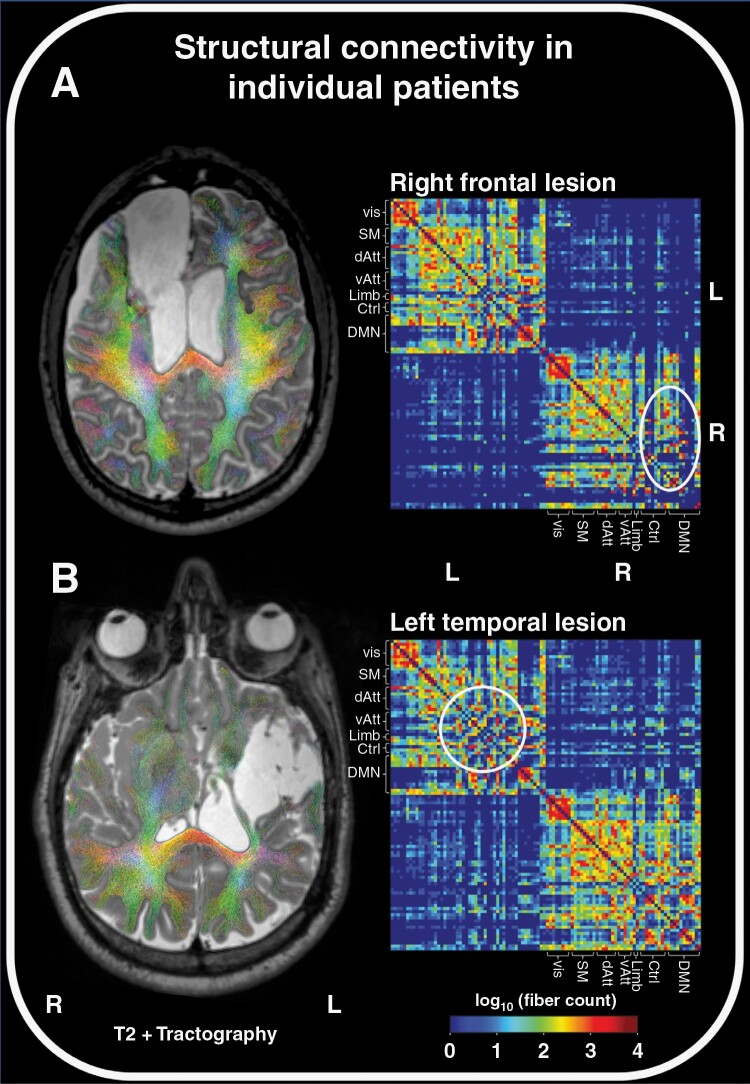
Whole-brain tractography and structural connectivity matrices in 2 exemplary patients with right frontal or left temporal gliomas. In the matrices, the log(10) of the fiber count connecting the respective cortical nodes belonging to 7 well-defined networks is color-coded as edge weight. White circles indicate sets of reduced connectivity. Vis, visual; SM, somatomotor; dAtt, dorsal attention; vAtt, ventral attention; Limb, limbic; Ctrl, frontal control; DMN, default mode network; L, left; R, right.

In order to obtain structural whole-brain connectivity matrices for each patient, the resulting set of fibers was combined with the functional cortical Schaefer-Yeo Atlas,^22^ which comprises 100 nodes (50 in each hemisphere) at its lowest resolution, belonging to the following 7 networks: visual, somatomotor, dorsal attention, ventral attention, limbic, frontal control, and default mode network. The number of fibers connecting any 2 nodes was used to enter a 100 × 100 structural connectivity matrix, thus representing the edge weight between 2 nodes. Whole-brain tractography and structural connectivity matrices for 2 exemplary patients with left frontal and right temporal gliomas are shown in Figure 2.

### Connectome-based Predictive Modeling

The relationship between structural brain connectivity and cognitive functions was analyzed using a well-established method (connectome-based predictive modeling [CPM]) initially described by Shen et al.^25^ that uses machine-learning methods and cross-validation to predict behavioral outcomes from brain connectivity measures. CPM has been proven to perform equally or better compared to many existing approaches in brain-behavior prediction.^25^ Also, compared to other machine-learning models, it has the advantages that it makes only use of linear operations, is purely data-driven, and can be clearly interpreted.

The CPM protocol comprises 4 steps which were performed in Matlab (Matlab R2022a, MathWorks, Natick, MA, USA). The structural connectivity matrices and corresponding cognitive test scores served as inputs. The main diagonal containing 100 meaningless entries was removed from the matrices for all further steps, leaving ([100 × 100]−100) = 9900 valid entries. For feature selection (i), each fiber count (edge weight) in the connectivity matrix was related to any of the cognitive test scores using Spearman’s rank correlation, and only significant (*P* < .001) edges were selected. Next, summary connectivity values (ii) were calculated from the selected edges by separately summing the fiber counts of edges with negative or positive associations with the cognitive scores. For model construction (iii), linear regressions between the cognitive scores and the summary connectivity scores were computed. Furthermore, the relation between demographic, clinical, histomolecular and other tumor-related variates and cognitive performance was evaluated at this step by nonparametric statistical methods: age, education, time since treatment initiation, number of surgical interventions, number of radiotherapy series, number of chemotherapy courses, lesion volumes (Spearman rank correlation); sex, IDH mutation status (wildtype vs mutant), glioma Grade 3 versus Grade 4, presence of a recurrent tumor (Mann–Whitney *U*-Test); tumor location (Kruskal–Wallis analysis of variance). Univariate and multivariate models were constructed where the significant variables from this analysis were included in the CPM model as confounding covariates. Finally, the model’s generalizability and predictive power (iv) were evaluated by leave-one-out cross-validation. The cognitive scores for each single patient were predicted using the described feature (edge) selection method and linear regression from the data of the remaining patients. The predicted scores were then compared to the patient’s actual scores using both Pearson correlation and a permutation test with 100 iterations.

### Brain Mapping of Edges and Nodes

As an additional step, binary matrices were constructed such that only those edges that correlated significantly with the cognitive scores in at least 90% of the cross-validation iterations (validated predictive edges) and had been included in the models with significant predictive power (*P* < .05) were labeled. For descriptive purposes, critically involved nodes of each network were identified from their degree, that is, the number of adjacent validated edges. The nonzero-degree-nodes’ degree distribution was used for setting thresholds for critically involved nodes (degree ≥ mean + 1× standard deviation) and highly critical nodes (degree ≥ mean + 2× standard deviation). In addition, all validated edges were classified according to their belonging to within- or between network connections. The nodes and their degrees were then visualized in their anatomic location using the Connectivity Viewer of the BioImage Suite Web 1.2.0 (https://bioimagesuiteweb.github.io/webapp/connviewer.html?species=human).

## Results

### Cognitive Performance

The detailed cognitive test scores in glioma patients and healthy individuals are shown in Supplementary Table 3. Glioma patients performed significantly lower than healthy individuals in all tests except for the Number Transcoding test. The highest deviation from the control group was observed in trail-making tests (TMT-A, time needed: +53.1%; TMT-B, time needed: +72.4%), followed by the semantic word fluency test (Imagined Shopping Tour, number of items: −24.6%). The lowest deviation was observed in a test on verbal working memory (Digit Span Forward, items: −7.5%). Depending on the test applied, 10%–47% of the patients were prone to a clinically relevant deficit.

### Connectome-based Predictive Modeling

As shown in Figure 2, the edge values (fiber counts) were clustered within the ipsilateral nodes of the different networks such that intra-hemispheric connectivity was more pronounced than inter-hemispheric connectivity. The number of connecting fibers of each node to any ipsi- or contralateral nodes spanned a wide range but was nearly equally distributed in the right (median fiber number, 48; range, 0–4462 fibers) compared to the left (median fiber number, 43; range, 0–4604 fibers) hemisphere. In lesions of both sides, the median nodal fiber count for intra-hemispheric connections was lower in the affected than in the contralateral hemisphere: median fiber number in left-sided lesions, 41 (range, 0–4035 fibers) versus 52 (range, 0–4849 fibers); median fiber number in right-sided lesions, 40 (range, 0–4013 fibers) versus 47 (range, 0–5265 fibers), representing an average fiber loss of 15%–20% per node in the affected hemisphere.

In the first step of the CPM analysis, 2770 node-to-node fiber counts with a significant correlation to any of the 10 cognitive test scores (predictive edges) were identified. In the vast majority (2704/2770 = 98%) of node-to-node fiber counts, the sign of the correlation indicated a positive association between fiber counts and cognitive scores (negative sign for the TMT-A and TMT-B, positive sign for all other tests, see Supplementary Table 4). A median number of 254 of the positively associated predictive edges per cognitive test (range, 32–542) was selected for linear regression modeling between the connectivity values and the corresponding cognitive performance scores of all 121 patients. All linear relationships were significant (*P* < .001), and most of them had a coefficient of determination (R^2^) in the range between 0.35 and 0.45, examples shown in Supplementary Figure 1. Conversely, models built from the negatively associated edges had R^2^-values in the range of 0.02 to 0.16 (Supplementary Table 4). The analysis of the demographic, clinical, or tumor-related variables showed significant (*P* < .01) relations with cognitive scores for age (9 of 10 scores), education (8 of 10 scores), tumor location (3 of 10 scores), lesion volumes (1–5 of 10 scores depending on lesion type) and tumor recurrence (1 of 10 scores), but no significant associations for sex, tumor grade, IDH-status, interval, surgical procedures, radiotherapy series, or chemotherapy courses (Supplementary Table 5).

**Figure 3.**
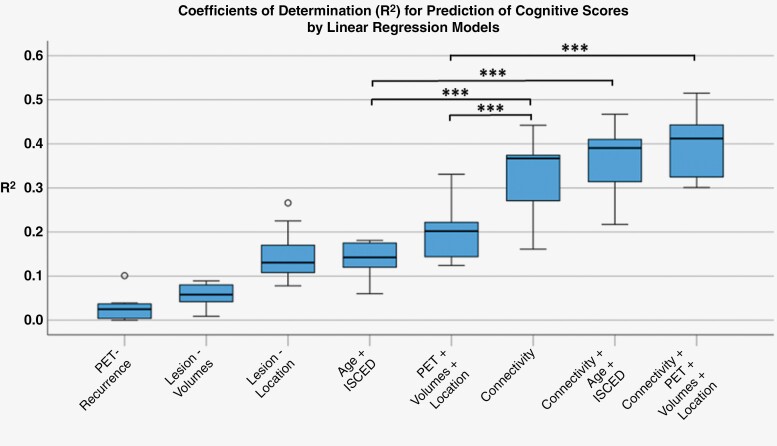
Performance of different linear regression models used for prediction of cognitive scores. The distributions of the coefficients of determination achieved in 10 cognitive tests are depicted as boxplots. The CPM-model using only the connectivity values outperformed the models using demographic and tumor-related variates, and the connectivity values significantly improved the performance of the latter models when additionally included. ISCED, International Standard Classification of Education; ***P < .001.

The predictive abilities of different models including the above-mentioned variates alone or in combination with the positively associated summary connectivity values are shown in Figure 3 and Supplementary Table 6. The pure connectivity models had a significantly higher mean coefficient of determination (0.330 ± 0.083) than the combined models from either age and education (0.141 ± 0.037) or recurrence and lesion volumes and tumor location (0.208 ± 0.071; both *P* < .001, *t*-test). The coefficient of determination of the latter models increased significantly by including the connectivity values (mean increase by 0.190 and 0.225, respectively; both *P* < .001), and all connectivity values proved their independent relation with the cognitive scores (all *P* < .001) in the combined multiple regression models.

**Figure 4.**
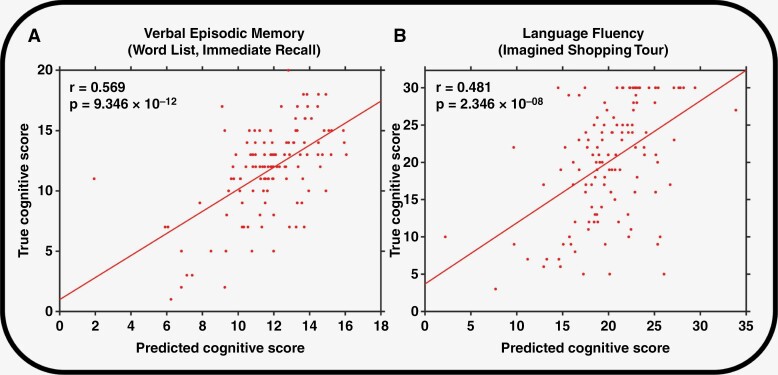
Correlation analysis between the predicted and actual scores of cognitive tests for verbal episodic memory (immediate recall) and language fluency in 121 glioma patients. *r*, correlation coefficient.

Therefore, the edges with a positive association to cognitive scores were exclusively used for the final, cross-validated model. The results for the correlation and permutation analyses between the predicted scores from the leave-one-out cross-validation and the actual scores are shown in Table 1 and Figure 4. The models predicted 7 out of 10 scores (median correlation coefficient, 0.47; range, 0.39–0.57) from 64 to 530 of 9900 (0.6%–5.4%) of the possible edges, underpinning the predictive value and potential generalizability of the developed model; illustrative examples are shown in Figure 4. However, the final model did not accurately predict the scores for the digit span tests evaluating the verbal working memory.

**Table 1.** Results of Model Cross-validation (CV)

	Model cross-validation (CV)^*^				Identification of validated edges^†^		
Test (cognitive domains)	Average edge number included (%)^‡^	RMSE	Correlation coefficient (*r*)	*P*-value permutation	Validated intra-network edges	Validated inter-network edges	Total validated edges (%)^‡^
Trail-making Test A (s)	514 (5.2%)	31.61	0.388***	0.01*	82	344	426 (4.3%)
(Attention, processing speed)							
Trail-making Test B (s)	530 (5.4%)	71.66	0.470***	0.01*	74	386	460 (4.6%)
(Executive function, concept shifting)							
Imagined shopping tour [items]	205 (2.1%)	6.82	0.481***	0.01*	26	148	174 (1.8%)
(Language, semantic word fluency)							
Number transcoding [items]	64 (0.6%)	0.99	0.411***	0.01*	10	38	48 (0.5%)
(Language processing)							
Digit span Fw [weighted items]	30 (0.3%)	2.43	0.123	0.13	CV not passed	CV not passed	CV not passed
(Verbal working memory)							
Digit span Bw [weighted items]	56 (0.6%)	2.58	0.132	0.16	CV not passed	CV not passed	CV not passed
(Verbal working memory)							
Corsi block tapping Fw [weighted items]	224 (2.3%)	2.32	0.202*	0.11	CV not passed	CV not passed	CV not passed
(Visuospatial working memory)							
Corsi block tapping Bw [weighted items]	319 (3.2%)	1.99	0.433***	0.01*	56	208	264 (2.7%)
(Visuospatial working memory)							
Word list, immediate recall [items]	440 (4.4%)	3.08	0.570***	0.01*	38	336	374 (3.8%)
(Verbal episodic memory)							
Word list, delayed recall [items]	272 (2.7%)	2.38	0.525***	0.01*	36	200	236 (2.4%)
(Verbal episodic memory)							

*Notes*: Fw = Forward; Bw = Backward; RSME = Root (of) Mean Squared Error.

^*^Models based on edges positively associated with cognitive performance.

^†^Edges significant in 90% of cross-validation iterations.

^‡^No edges from the nodes to themselves, *n* = ([100 × 100]–100) = 9900 possible edges.

*P < .05. ***P < .001.

### Brain Mapping of Validated Edges and Critical Nodes

**Figure 5.**
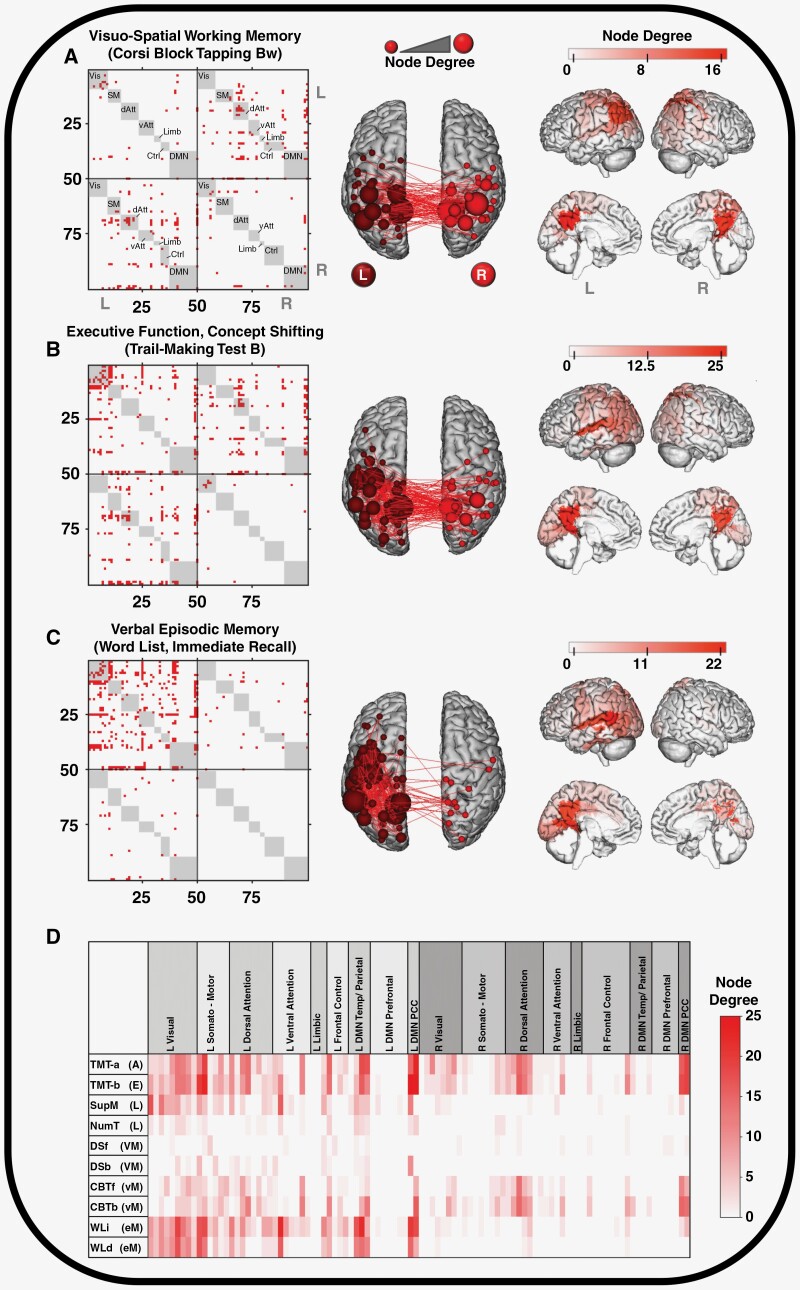
Left: Binary connectivity matrices labeling cross-validated predictive edges; membership of nodes to networks marked in gray. Middle/Right: Anatomical representation of the critical nodes by visualization of node degree and connecting edges. Results are shown for 3 representative cognitive tests. (**A**) Corsi Block Tapping (visual working memory), (**B**) Trail-making Test B (processing speed), (**C**) Word List, immediate recall (verbal semantic memory). (**D**) Heatmap of the networks and nodes and their degrees of adjacent cross-validated predictive edges concerning the raised cognitive scores. Bw, Backward; L, left; R, right; Vis, visual; SM, somatomotor; dAtt, dorsal attention; vAtt, ventral attention; Limb, limbic; Ctrl, frontal control; DMN, default mode network; Temp, temporal; TMT-A (A), Trail-Making Test A (attention); TMT-B (E), Trail-Making Test B (executive function); SupM (L), Imagined Shopping Tour (language); DSf (VM), Digit Span Forward (verbal working memory); DSb (VM), Digit Span Backward (verbal working memory); CBTf (vM), Corsi Block Tapping Forward (visuospatial working memory); CBTb (vM), Corsi Block Tapping Backward (visuospatial working memory); WLi (eM), Word List, immediate recall (verbal episodic memory); WLd (eM), Word List, delayed recall (verbal episodic memory); PCC, posterior cingulate cortex.

The binary matrices of the cross-validated edges for some cognitive tests are shown in Figure 5A–C and Supplementary Figure 2 together with an anatomical representation of the validated edges and their adjacent nodes. The validated edges followed a pattern of mainly left intra-hemispheric as well as inter-hemispherical connections. Of note, the majority (overall 1660/1982 = 84%; Table 1) of the validated edges were between nodes of different networks (inter-network connections, median 208, range 38–386) rather than between nodes of the same network (median 19, range 5–41). This observation is also evident from the binary matrices (Figure 5A–C, Supplementary Figure 2) where all potential intra-network connections are displayed in gray. The few negatively associated edges (identified in Step 1) were sparsely distributed with a tendency to be situated between nodes of the right hemisphere (Supplementary Figure 3).

A detailed heatmap of the network nodes and their degrees of adjacent validated edges concerning the raised cognitive scores are shown in Figure 5D. The nodes adjacent to a nonzero number of validated edges had a degree of 5.4 ± 5.2; therefore, nodes with a degree ≥10 validated edges were regarded as critical for descriptive purposes here. The distribution of these critically involved nodes varied considerably between domains. Critical nodes for attention/processing speed (TMT-A) and executive function/concept shifting (TMT-B) were mainly located in the left visual and somatomotor networks but also included nodes from the bilateral dorsal attention and default mode networks. For performance in visual working memory (Corsi Block Tapping), mainly nodes of the right dorsal attention and bilateral default mode networks proved critical and were predominantly connected by inter-hemispherical fibers. Nodes and connections critically involved in verbal semantic memory (Word List, immediate and delayed recall) were almost exclusively left-sided and included nodes from the visual, somatomotor, and default mode networks (Figure 5A–C; Supplementary Figure 2). Interestingly, highly critical nodes (degree ≥15 adjacent validated edges) included the default mode network nodes in the left temporal/parietal or bilateral posterior cingulate cortex in 4 to 5 of the 7 predictive models.

## Discussion

As demonstrated in recent randomized trials and the present study, glioma patients are at high risk of developing cognitive decline during their course of disease.^3^ Although the relationship between brain damage and neurological function is generally well established for the eloquent primary cortical areas and their associated fiber tracts,^35^ the underlying causes of cognitive deterioration in brain tumor patients remain poorly understood.^36–38^ Nevertheless, initial studies in glioma patients suggested that evaluating brain networks may help further elucidate the cognitive decline of various domains.^13–15,39,40^ In the present study, we hypothesized that decreased structural connectivity in whole-brain networks is associated with cognitive deterioration in glioma patients. Therefore, we applied an innovative tractography algorithm^4,23^ in combination with a network-based parcellation that allowed the construction of a whole-brain connectome of the structurally altered brain of pretreated glioma patients in conjunction with a well-developed method for predicting traits and symptoms from connectivity data.^25^ Thus, we could show that reduced fiber numbers in subsets of connections mainly connecting different brain networks were significantly related to performance deficits in different cognitive domains. Critical cortical regions (nodes) having cross-validated connections to a high number of other nodes included mainly left-hemispheric cortical regions nodes and several cortical regions known as hubs, such as the bilateral precuneus or posterior cingulate cortex.^41^

As expected, lesion location concerning the major cerebral lobes was significantly associated with reduced scores in a subset of cognitive tests. Of note, this only gives a rough orientation and does not allow for a fine-grained characterization of the cortical regions and fiber tracts involved in the performance of specific cognitive domains. In principle, the relation between lesion location and symptom severity could have been brought down to the voxel level, resulting in the widely used method of voxel-based lesion-symptom mapping which has also been applied in brain tumors.^42^ However, despite the high spatial resolution, this method has the disadvantage that it arbitrarily maps gray and white matter and can only be applied in brain locations with a representative number of lesions. This may result in diverging results depending on the pathology under investigation, for example, for tumors versus stroke.^43^

### Whole-brain Connectome: The Importance of Networks and Hubs

Although the integrity of single fiber tracts appears to have a measurable impact on different aspects of cognitive functioning, most higher brain functions are probably supported by more general organizational principles governing the information flow in the brain. Regarding structural connectivity, several highly connected cortical regions have been identified and termed the “rich club.”^41^ Most of these are also present in functional resting-state networks (RSN)^44,45^ and are predominantly located in the posterior part of the default mode network.^45,46^ Functionally, they seem to serve primarily for connectivity between the RSNs, especially between the default mode, attention, and control networks.^45^

These findings and the availability of advanced MR imaging techniques in the clinic have led to more network-oriented approaches for investigating the dependence of cognitive performance on structural connectivity in perioperative glioma patients. In patients with low-grade gliomas, Cocherau et al.^17^ studied a more extensive set of tracts using tract-wise lesion-symptom mapping and found the integrity of the left superior longitudinal fascicle and left frontal aslant/frontostriatal tracts to be most predictive for the development of postoperative disturbances in executive functioning and phonologic fluency. Liu et al.^18^ constructed a whole-brain structural connectome from deterministic tractography in preoperative glioma patients. They observed that local (node) efficiency, a measure for communication strength among the first neighbors of a node, was generally reduced in tumor patients and particularly related to memory function in temporal tumors and to information processing speed in frontal tumors. Zhang et al.^16^ applied navigated transcranial magnetic stimulation (nTMS) and whole-brain deterministic tractography in left-sided tumors and observed a correlation between the average node degree (and other connectome properties) of the left-hemispheric/nTMS-positive networks and the degree of aphasia. A network-level approach was also adopted by Mrah et al.,^19^ who computed lesion overlap and white matter dysconnectivity scores for several atlas-based functional networks in low-grade glioma. Through a machine-learning algorithm, lesions or disconnections of the frontoparietal (control) network proved to be most predictive for postoperative deterioration in cognitive set-shifting.

In the present study, we used a network and node definition scheme encompassing the entire cerebral cortex and considered all potential structural connections between cortical nodes, including those lying outside anatomically designated tracts. Despite significant variation between cognitive domains, most predictive connections were those between different RSNs rather than within single RSNs. The distribution of critically involved nodes also varied considerably between domains but included nodes of the left visual and somatomotor networks and bilateral nodes of the dorsal attentional and default mode networks in several domains. Particularly critical nodes included the default mode network’s left temporal and bilateral posterior cingulate cortex. These findings fit well with the view that structural connections between RSNs form the backbone of functional connectivity, enabling higher cognitive processes. From a more general point of view, these results may imply that cognitive decline in treated brain tumor patients shares a common mechanism with other major psychiatric and neurological disorders where the rich club nodes were also found to be predominantly involved,^47^ such as the precuneus/posterior cingulate cortex in Alzheimer’s disease.^48^

As cognitive performance depends on several nodes of different networks, the present models could be used to predict cognitive decline in individual glioma patients especially when local treatments such as surgery and radiotherapy are planned. In these situations, post-therapeutic cognitive deficits could arise unforeseen by clinical judgment or standard neuro-navigation, but may be anticipated or avoided by pre-therapeutic whole-brain tractography and critical node definitions as provided here.

### Limitations

This study included patients with substantial variability in treatment intensity and time between treatment initiation and imaging/neurocognitive assessment. In addition, each patient was observed only once, such that longitudinal observations were not available. On the other hand, while group analyses are usually challenging to perform in this constellation, a rich, diverse pattern of structural damage may have facilitated the construction of a predictive model for cognition performance. From a methodological point of view, applying an atlas-based parcellation created from healthy subjects may be questionable because a functional restructuring of the brain, including shifts and deformations of cortical nodes, may have occurred in the patients. Finally, fiber tractography always approximates the actual white matter structure because even the most advanced methods may fail, especially in structurally altered brain tissue.

## Conclusion

In summary, the present results suggest that the cognitive performance of pretreated glioma patients is strongly related to the structural connectivity between multiple brain networks and the integrity of known network hubs. This mirrors a pattern observed for other major neurological disorders. Whole-brain tractography in conjunction with the definition of critical cortical nodes should be further evaluated for improving local treatment planning in glioma patients.

## Supplementary Material

vdad151_suppl_Supplementary_Tables_S1-S6_Figures_S1-S3

## Data Availability

Anonymized and aggregated data are available upon reasonable request.
