# Supplementary material for: Structural connectome-based predictive modeling of cognitive deficits in treated glioma patients
Source: Neurooncol Adv. 2023 Nov 15;6(1):vdad151. doi: 10.1093/noajnl/vdad151 (PMC10776208; doi:10.1093/noajnl/vdad151)
Supplement: vdad151_suppl_Supplementary_Tables_S1-S6_Figures_S1-S3 [file vdad151_suppl_supplementary_tables_s1-s6_figures_s1-s3.docx]

**Supplementary Material**

**Structural connectome-based predictive modeling of cognitive deficits in treated glioma patients**

Michel Friedrich^1^, Christian P. Filss^1^, Philipp Lohmann^1^, Felix M. Mottaghy^2^,
Gabriele Stoffels^1^, Carolin Weiss Lucas^3,4^, Maximilian I. Ruge^4,5^, N. Jon Shah^1,6,7^,
Svenja Caspers^1,8^, Karl-Josef Langen^1,2,4^, Gereon R. Fink^1,9^,
Norbert Galldiks^1,4,9†^, Martin Kocher^1,4,5†^

**^†^These authors contributed equally to senior authorship.**

**Author affiliations**

^1^Institute of Neuroscience and Medicine (INM-1, -3, -4, -11), Forschungszentrum Juelich, Juelich, Germany (MF, CPF, PL, GS, NJS, SC, KJL, GRF, NG, MK)

^2^Department of Nuclear Medicine, RWTH University Hospital Aachen, RWTH University Aachen, Aachen, Germany (FMM, KJL)

^3^Department of General Neurosurgery, Center for Neurosurgery, Faculty of Medicine and University Hospital Cologne, University of Cologne, Cologne, Germany (CWL)

^4^Center of Integrated Oncology (CIO), Universities of Aachen, Bonn, Cologne, and Duesseldorf, Germany (CWL, MIR, KJL, NG, MK)

^5^Department of Stereotaxy and Functional Neurosurgery, Center for Neurosurgery, Faculty of Medicine and University Hospital Cologne, Cologne, Germany (MIR, MK)

^6^Juelich-Aachen Research Alliance (JARA), Section JARA-Brain, Juelich, Germany (NJS)

^7^Department of Neurology, RWTH University Hospital Aachen, RWTH University Aachen, Aachen, Germany (NJS)

^8^Institute for Anatomy I, Medical Faculty and University Hospital Duesseldorf, Heinrich Heine University Duesseldorf, Duesseldorf, Germany (SC)

^9^Department of Neurology, Faculty of Medicine and University Hospital Cologne, University of Cologne, Cologne, Germany (GRF, NG)

**Table S1** Patient characteristics

|  | |  |  |
| --- | --- | --- | --- |
| **Sex (male/female)** | | 73/48 |  |
| **Age (years)** | | 52 | (28-74) |
| **ECOG-PS (0/1/2/3)** | | 58/56/6/1 |  |
| **Education (ISCED-Score)** | | 8 | (3-10) |
| **Employment (no/yes)** | | 45/76 |  |
| **Follow-up Interval (months)** | | 14.4 | (0.6-213.7) |
|  | |  |  |
| **Presenting Symptoms** | |  |  |
| Aphasia | | 17 | (14%) |
| Paresis | | 29 | (24%) |
| Fatigue | | 19 | (16%) |
| Vision disturbance | | 12 | (10%) |
| Vertigo, Confusion | | 4 | (3%) |
|  | |  |  |
| **WHO CNS 2021 Tumor Type** | |  |  |
| GBM, IDH wildtype | | 72 | (60%) |
| GBM, NOS | | 4 | (3%) |
| Astrocytoma, IDH-mutated Grade 4 | | 8 | (7%) |
| Astrocytoma, IDH-mutated Grade 3 | | 15 | (12%) |
| Astrocytoma, NOS Grade 3 | | 6 | (5%) |
| Oligodendroglioma, IDH-mutated, 1p19q codeleted G3 | | 13 | (11%) |
| Oligodendroglioma NOS/NEC Grade 3 | | 3 | (2%) |
|  | |  |  |
| **Tumor Location^a^** | |  |  |
| Frontal Left/Right | | 31/28 | (26%/23%) |
| Parietal Left/Right | | 7/8 | (6%/7%) |
| Temporal Left/Right | | 22/16 | (18%/13%) |
| Occipital Left/Right | | 5/4 | (4%/3%) |
|  | |  |  |
| **Lesion Volumes (mL)** | |  |  |
| Resection cavity | (n= 90) | 20.9 | (0.3-172.5) |
| FLAIR hyperintense | (n=121) | 53.4 | (3.4-252.9) |
| T1 contrast-enhancing | (n= 99) | 8.3 | (0.01-122.9) |
| ^18^F-FET PET (TBR > 1.6) | (n= 79) | 30.3 | (2.6-227.8) |
|  | |  |  |
| **Treatment (Number of Procedures)** | |  |  |
| Surgery^b^ | (1/2/3/4) | 101/17/2/1 |  |
| Radiotherapy | (0/1/2) | 7/100/14 |  |
| Chemotherapy | (0/1/2/3) | 10/91/16/4 |  |
|  | |  |  |
| **Corticosteroids (no/yes)** | | 91/30 | (75%/25%) |
| **Anticonvulsants (no/yes)** | | 49/72 | (40%/60%) |
| **^18^F-FET PET diagnosed recurrence (no/yes)** | | 63/58 | (52%/48%) |
|  | |  |  |

Median (Range) unless otherwise stated; ECOG-PS, Eastern Cooperative Oncology Group Performance Score; ISCED, International Standard Classification of Education (1997); GBM, glioblastoma; IDH, Isocitrate-Dehydrogenase; NOS, not otherwise specified; NEC, not elsewhere classified; AA, anaplastic astrocytoma; AOD, anaplastic oligodendroglioma; 1p19q codel, 1p19q co-deleted; ^a^main lobe involved; FLAIR, fluid-attenuated inversion recovery; ^18^F-FET, O-(2-[^18^F]fluoroethyl)-L-tyrosine; TBR, tumor-to-brain ratio; ^b^including biopsy

**Table S2** Description of Neurocognitive Tests

All tests were done with paper and pencil. The tests are shown here in the order in which they were presented to the patient.

- **Trail-Making Test A**: This widely used test assesses processing speed and attention. Small circles enclosing the numbers 1-25 are randomly distributed on a sheet of paper, and the patient is instructed to draw a connecting line in ascending order as quickly as possible without lifting the pencil. The time for completion is measured and given in seconds.^1,2^

- **Trail Making Test B**: This is a variant of the Trail-Making Test A, which measures executive function and concept shifting. Small circles with the numbers 1-13 and the letters A-L are randomly distributed on a sheet of paper, and the patient has to connect them in ascending, alternating order as quickly as possible, measured in seconds.^1,2^

- **Digit Span forward**: This is a common test for verbal working memory.^3^ Increasingly long lists of number words are read to the patient, who is asked to recall each list immediately from memory. Two points are awarded for success on the first attempt. In the event of a failed attempt, another list of the same length is read to the patient, for which she receives one point if recalled correctly. After two failed attempts, the test is stopped, and the total score is counted.

- **Word List, immediate recall**: This test is part of the DemTect^4^ battery widely used for dementia screening. A list of 10 items is read to the patient, who is instructed to recall as many items as possible immediately after presentation. The same list is then presented a second time, and the total number of correctly recalled items from these two runs (up to 20) is counted. No instruction is given to remember the items for later recall.

- **Imagined Shopping Tour**: This test is also part of the DemTect and is used to assess semantic word fluency. The patient is instructed to imagine a shopping trip in a supermarket, imagining and pronouncing as many products as possible within 60 seconds. The total number of items is counted (up to 30).

- **Number Transcoding**: This test is also part of the DemTect battery and tests aspects of language processing. Two numbers presented in numerical notation have to be transformed into whole words, and two numbers written in whole words have to be transformed to numerical notation. The number of successes (out of four) is counted.

- **Digit Span backward**: Same as Digit Span forward, but with the instruction to recall the list of numbers in reverse sequence.

- **Word List, delayed recall**: The patient is asked to recall the list of 10 items from the word list presented before (typical interval of 5-10 minutes), maximum score of 10 items.

**- Corsi Block Tapping Test:** This is a test for visual-spatial working memory.^5^ The examiner taps increasingly long sequences of 10 small blocks mounted on a board, and the patient is asked to reproduce the sequence forward/backward by tapping the blocks himself. The same scoring scheme as for the digit span tests is applied.

**Table S3** Cognitive test scores in glioma patients and healthy subjects

| **Test**  (Cognitive Domains) | **Glioma Patients** | **Healthy Subjects** | **Difference (%)** | **Patients (%) with Clinically Relevant Deficit^a^** |
| --- | --- | --- | --- | --- |
|  |  |  |  |  |
| **Trail-Making Test A [seconds]** | 47.3 ± 33.9 *** | 30.9 ± 12.1 | +53.1 | 39 (32%) |
| (Attention, processing speed) |  |  |  |  |
|  |  |  |  |  |
| **Trail-Making Test B [seconds]** | 117.6 ± 80.2 *** | 68.2 ± 40.1 | +72.4 | 34 (28%) |
| (Executive function, concept shifting) |  |  |  |  |
|  |  |  |  |  |
| **Imagined Shopping Tour [items]** | 20.2 ± 7.7 *** | 26.8 ± 4.4 | -24.6 | 57 (47%) |
| (Language, semantic word fluency) |  |  |  |  |
|  |  |  |  |  |
| **Number Transcoding [items]** | 3.3 ± 1.1 n.s. | 3.6 ± 0.6 | -8.3 | 21 (17%) |
| (Language processing) |  |  |  |  |
|  |  |  |  |  |
| **Digit Span Forward [weighted items]** | 7.4 ± 2.3 ** | 8.0 ± 2.3 | -7.5 | 12 (10%) |
| (Verbal working memory) |  |  |  |  |
|  |  |  |  |  |
| **Digit Span Backward [weighted items]** | 6.5 ± 2.5 *** | 8.3 ± 2.3 | -21.7 | 20 (17%) |
| (Verbal working memory) |  |  |  |  |
|  |  |  |  |  |
| **Corsi Block Tapping Fw [weighted items]** | 6.6 ± 2.3 * | 7.4 ± 1.9 | -10.8 | 27 (22%) |
| (Visuo-spatial working memory) |  |  |  |  |
|  |  |  |  |  |
| **Corsi Block Tapping Bw [weighted items]** | 4.8 ± 2.2 *** | 6.0 ± 2.0 | -20.0 | 28 (23%) |
| (Visuo-spatial working memory) |  |  |  |  |
|  |  |  |  |  |
| **Word List, Immediate Recall [items]** | 11.7 ± 3.7 *** | 14.1 ± 2.6 | -17.0 | 34 (28%) |
| (Verbal episodic memory) |  |  |  |  |
|  |  |  |  |  |
| **Word List, Delayed Recall [items]** | 4.5 ± 2.8 * | 5.4 ± 2.4 | -16.7 | 22 (18%) |
| (Verbal episodic memory) |  |  |  |  |
|  |  |  |  |  |

Fw, Forward; Bw, Backward; ^a^below the mean - 1.5 x the standard deviation of healthy subjects.
* p < 0.05, ** p < 0.01, *** p < 0.001, two-sided Mann–Whitney U-test

**Table S4** Coefficients of determination (R^2^) of univariate linear regression analysis for cognitive scores of the entire patient group. Summary connectivity values served as predictors and were calculated separately for edges positively and negatively associated with cognitive performance.

|  | **Edges positively associated with cognitive performance** | | **Edges negatively associated with cognitive performance** | |
| --- | --- | --- | --- | --- |
| **Test** (Cognitive Domains) | **Edge Number** | **Coefficient of Determination (R^2^)** | **Edge Number** | **Coefficient of Determination (R^2^)** |
|  |  |  |  |  |
|  |  |  |  |  |
| **Trail-Making Test A** | 524 | 0.255 *** | 16 | 0.070 ** |
| (Attention, processing speed) |  |  |  |  |
|  |  |  |  |  |
| **Trail-Making Test B** | 542 | 0.365 *** | 8 | 0.086 ** |
| (Executive function, concept shifting) |  |  |  |  |
|  |  |  |  |  |
| **Imagined Shopping Tour** | 206 | 0.374 *** | 6 | 0.093 ** |
| (Language, semantic word fluency) |  |  |  |  |
|  |  |  |  |  |
| **Number Transcoding** | 66 | 0.369 *** | 6 | 0.154 *** |
| (Language processing) |  |  |  |  |
|  |  |  |  |  |
| **Digit Span Forward** | 32 | 0.161 *** | 0 | ------- |
| (Verbal working memory) |  |  |  |  |
|  |  |  |  |  |
| **Digit Span Backward** | 56 | 0.271 *** | 4 | 0.024 |
| (Verbal working memory) |  |  |  |  |
|  |  |  |  |  |
| **Corsi Block Tapping Forward** | 226 | 0.294 *** | 0 | ------- |
| (Visuo-spatial working memory) |  |  |  |  |
|  |  |  |  |  |
| **Corsi Block Tapping Backward** | 316 | 0.370 *** | 12 | 0.158 *** |
| (Visuo-spatial working memory) |  |  |  |  |
|  |  |  |  |  |
| **Word List, Immediate Recall** | 454 | 0.442 *** | 2 | 0.128 *** |
| (Verbal episodic memory) |  |  |  |  |
|  |  |  |  |  |
| **Word List, Delayed Recall** | 282 | 0.398 *** | 12 | 0.107 *** |
| (Verbal episodic memory) |  |  |  |  |
|  |  |  |  |  |

** p < 0.01, *** p < 0.001

**Table S5** Clinical factors mainly unrelated to cognitive test scores in treated glioma patients

| **Test**  (Cognitive Domains) | **Sex (MWU p)** | **Tumor Grade (MWU p)** | **IDH-status (MWU p)** | **Interval (Spearman rho)** | **Pretreatment Surgery**  **(Spearman rho)** | **Pretreatment Radiotherapy**  **(Spearman rho)** | **Pretreatment Chemotherapy**  **(Spearman rho)** |
| --- | --- | --- | --- | --- | --- | --- | --- |
|  |  |  |  |  |  |  |  |
| **Trail-Making Test A** | 0.167 | 0.045 | 0.014 * | - 0.175 | - 0.107 | - 0.001 | - 0.043 |
| (Attention, processing speed) |  |  |  |  |  |  |  |
|  |  |  |  |  |  |  |  |
| **Trail-Making Test B** | 0.159 | 0.223 | 0.101 | - 0.134 | - 0.090 | - 0.036 | - 0.003 |
| (Executive. function, concept shifting) |  |  |  |  |  |  |  |
|  |  |  |  |  |  |  |  |
| **Imagined Shopping Tour** | 0.361 | 0.464 | 0.969 | - 0.024 | - 0.038 | - 0.015 | - 0.088 |
| (Language, semantic word fluency) |  |  |  |  |  |  |  |
|  |  |  |  |  |  |  |  |
| **Number Transcoding** | 0.482 | 0.426 | 0.765 | - 0.051 | - 0.074 | - 0.047 | - 0.075 |
| (Language processing) |  |  |  |  |  |  |  |
|  |  |  |  |  |  |  |  |
| **Digit Span Forward** | 0.685 | 0.107 | 0.129 | - 0.031 | - 0.066 | - 0.083 | - 0.026 |
| (Verbal working memory) |  |  |  |  |  |  |  |
|  |  |  |  |  |  |  |  |
| **Digit Span Backward** | 0.564 | 0.153 | 0.190 | 0.214 * | 0.143 | 0.082 | 0.136 |
| (Verbal working memory) |  |  |  |  |  |  |  |
|  |  |  |  |  |  |  |  |
| **Corsi Block Tapping Forward** | 0.288 | 0.055 | 0.086 | - 0.005 | - 0.031 | - 0.163 | - 0.114 |
| (Visuo-spatial working memory) |  |  |  |  |  |  |  |
|  |  |  |  |  |  |  |  |
| **Corsi Block Tapping Backward** | 0.726 | 0.099 | 0.049 * | 0.041 | 0.042 | 0.044 | 0.015 |
| (Visuo-spatial working memory) |  |  |  |  |  |  |  |
|  |  |  |  |  |  |  |  |
| **Word List, Immediate Recall** | 0.477 | 0.574 | 0.518 | - 0.064 | 0.003 | - 0.018 | - 0.079 |
| (Verbal episodic memory) |  |  |  |  |  |  |  |
|  |  |  |  |  |  |  |  |
| **Word List, Delayed Recall** | 0.911 | 0.610 | 0.078 | - 0.014 | 0.054 | 0.034 | - 0.094 |
| (Verbal episodic memory) |  |  |  |  |  |  |  |
|  |  |  |  |  |  |  |  |
| **Number of tests with p < 0.01** | **0/10** | **0/10** | **0/10** | **0/10** | **0/10** | **0/10** | **0/10** |

* p < 0.05, ** p < 0.01, *** p < 0.001; MWU, two-sided Mann–Whitney U-test; ISCED, International Standard Classification of Education

**Table S6** Coefficients of determination (R^2^) of multiple linear regression models of cognitive scores for the entire patient group, using different combinations of predictors.

| **Test** (Cognitive Domains) | **PET- Recurrence** | **Lesion Volumes** | **Lesion Location** | **Age + ISCED** | **Recurrence + Volumes + Location** | **Summary Connectivity Values** | **Age + ISCED + Connectivity** | **PET-Recurrence + Volumes + Location + Connectivity** |
| --- | --- | --- | --- | --- | --- | --- | --- | --- |
| **Trail-Making Test A** | 0.039 * | 0.060 | 0.130 * | 0.131 *** | 0.201 * | 0.255*** | 0.291 *** | 0.331 *** |
| (Attention, processing speed) |  |  |  |  |  |  |  |  |
| **Trail-Making Test B** | 0.036 * | 0.052 | 0.080 | 0.179 *** | 0.144 | 0.365*** | 0.392 *** | 0.415 *** |
| (Executive function, concept shifting) |  |  |  |  |  |  |  |  |
|  |  |  |  |  |  |  |  |  |
| **Imagined Shopping Tour** | 0.003 | 0.080* | 0.155 ** | 0.118 ** | 0.222 ** | 0.374*** | 0.389 *** | 0.430 *** |
| (Language, semantic word fluency) |  |  |  |  |  |  |  |  |
|  |  |  |  |  |  |  |  |  |
| **Number Transcoding** | 0.009 | 0.009 | 0.131 * | 0.141 *** | 0.143 | 0.369 *** | 0.401 *** | 0.443 *** |
| (Language processing) |  |  |  |  |  |  |  |  |
|  |  |  |  |  |  |  |  |  |
| **Digit Span Forward** | 0.000 | 0.032 | 0.170 ** | 0.060 * | 0.212 ** | 0.161 *** | 0.217 *** | 0.301 *** |
| (Verbal working memory) |  |  |  |  |  |  |  |  |
| **Digit Span Backward** | 0.004 | 0.042 | 0.078 | 0.144 *** | 0.124 | 0.271 *** | 0.314 *** | 0.315 *** |
| (Verbal working memory) |  |  |  |  |  |  |  |  |
|  |  |  |  |  |  |  |  |  |
| **Corsi Block Tapping Forward** | 0.018 | 0.084* | 0.110 | 0.181 ** | 0.179 * | 0.294 *** | 0.343 *** | 0.325 *** |
| (Visuo-spatial working memory) |  |  |  |  |  |  |  |  |
| **Corsi Block Tapping Backward** | 0.101 *** | 0.056 | 0.108 | 0.175 *** | 0.203 * | 0.370 *** | 0.410 *** | 0.409 *** |
| (Visuo-spatial working memory) |  |  |  |  |  |  |  |  |
|  |  |  |  |  |  |  |  |  |
| **Word List, Immediate Recall** | 0.032 * | 0.089* | 0.225 *** | 0.120 ** | 0.325 *** | 0.442 *** | 0.467 *** | 0.515 *** |
| (Verbal episodic memory) |  |  |  |  |  |  |  |  |
| **Word List, Delayed Recall** | 0.037 | 0.073 | 0.266 *** | 0.165 ** | 0.331 *** | 0.398 *** | 0.436 *** | 0.493 *** |
| (Verbal episodic memory) |  |  |  |  |  |  |  |  |
|  |  |  |  |  |  |  |  |  |
| **Mean (Standard Deviation) of R^2^** | 0.028 (0.030) | 0.058 (0.025) | 0.145 (0.061) | 0.141 (0.037) | 0.208 (0.071) | 0.330 (0.083) | 0.366 (0.075) | 0.398 (0.076) |
| **p(Connectivity) in Combined Models** |  |  |  |  |  |  | all < 0.001*** | all < 0.001*** |

* p < 0.05, ** p < 0.01, *** p < 0.001; ISCED, International Standard Classification of Education (1997)


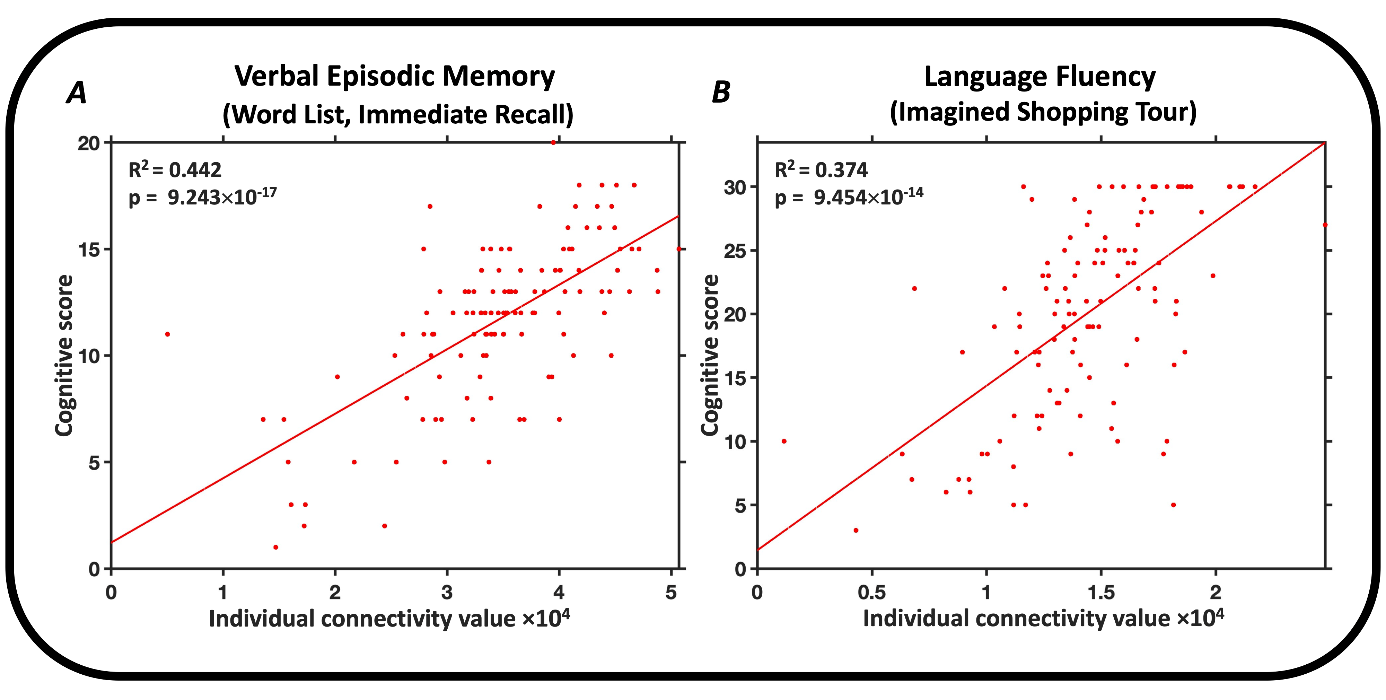


**Figure S1** Linear regression analysis for the dependency of cognitive test scores for verbal episodic memory (immediate recall) and language fluency on the individual connectivity scores (summed fiber counts) in 121 glioma patients. R^2^, coefficient of determination.


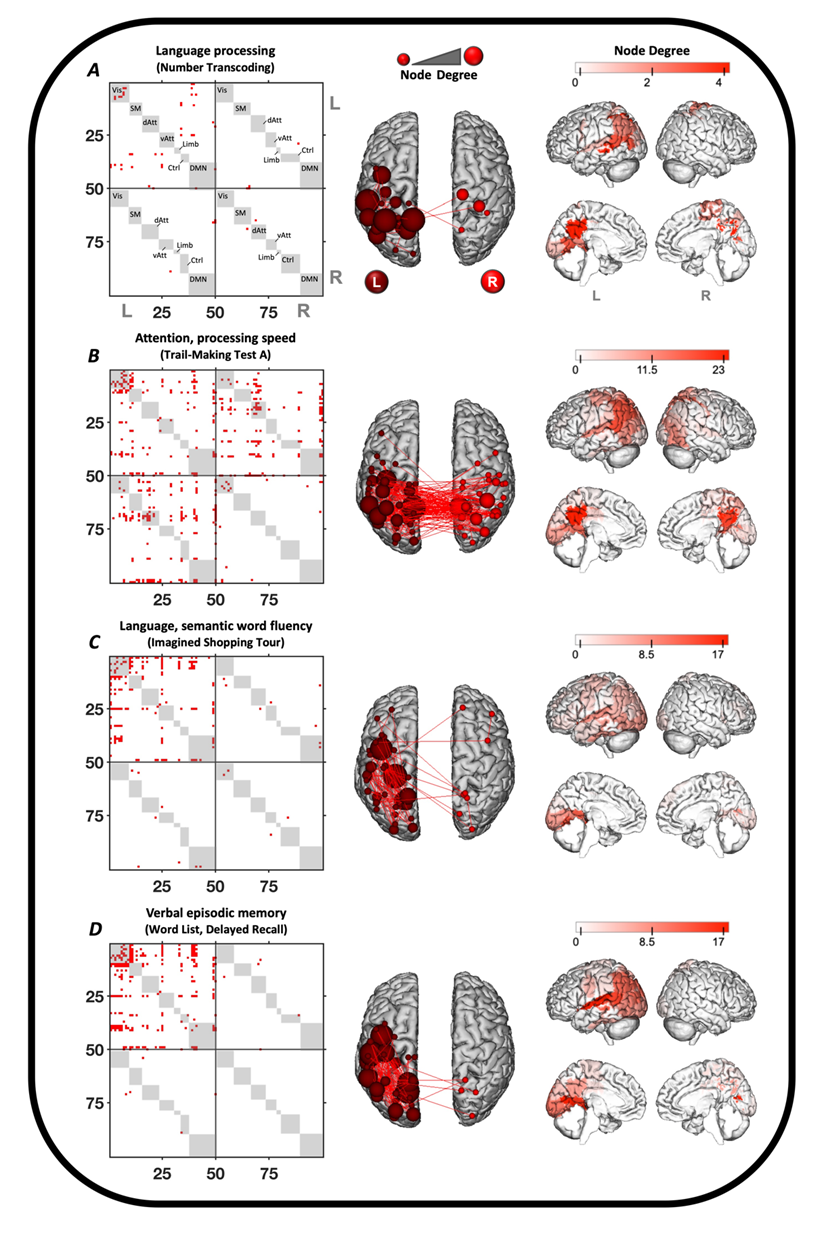


**Figure S2** Left: Binary connectivity matrix labeling cross-validated predictive edges; node membership to networks marked in gray. Middle/Right: Anatomical representation of the critical nodes by visualization of node degree and connecting edges. Results are shown for four representative cognitive tests. **A:** Number transcoding (language processing), **B:** Trail-Making Test A (attention/processing speed), **C:** Imagined Shopping Tour (semantic word fluency), **D:** Word List, delayed recall (verbal episodic memory). Vis, visual; SM, somatomotor; dAtt, dorsal attention; vAtt, ventral attention; Limb, limbic; Ctr, frontal control; DMN, default mode network


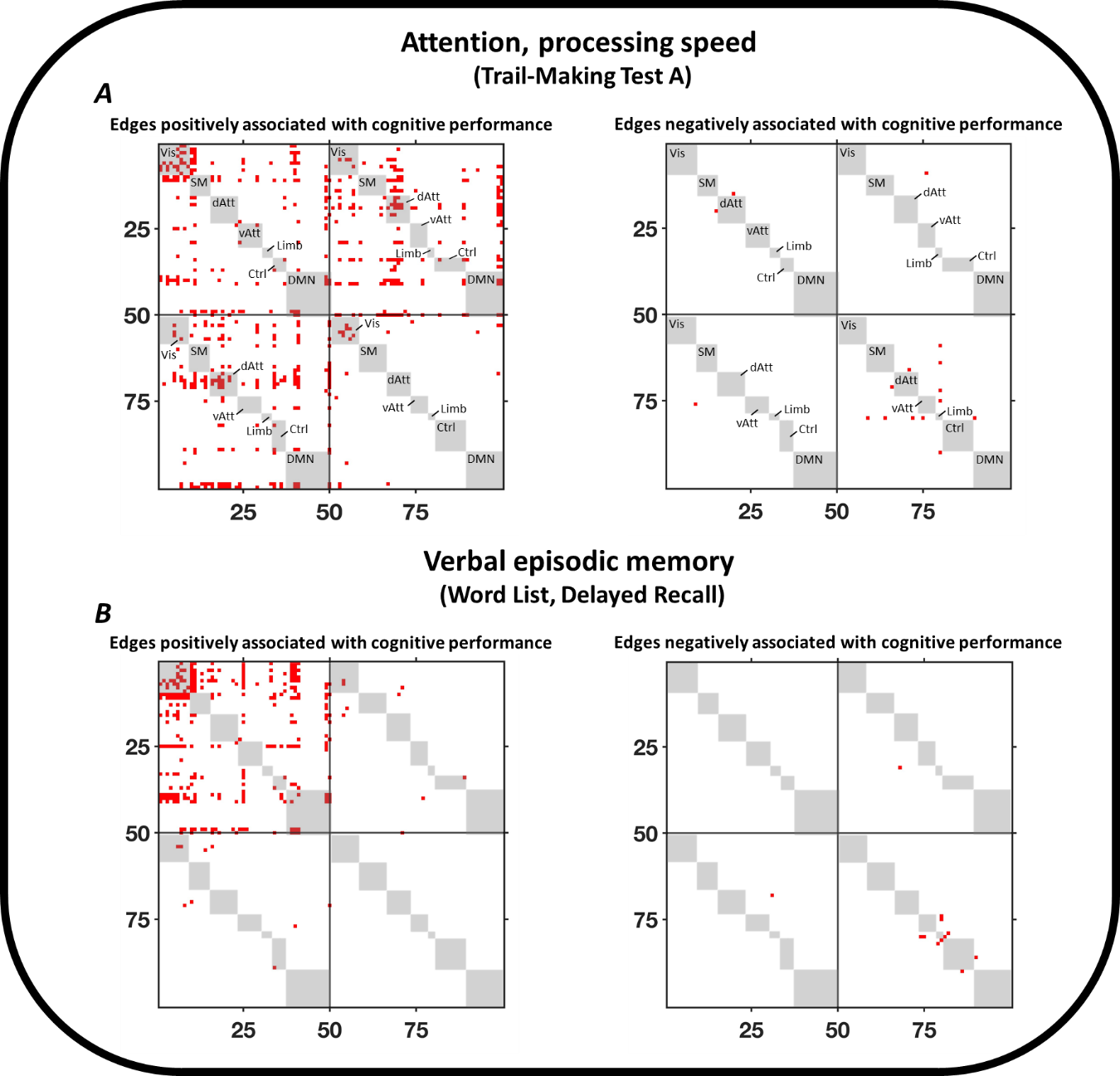


**Figure S3** Binary connectivity matrices labeling predictive edges for two exemplary tests of **A**) Attention/processing speed (Trail-Making Test A) and **B**) Verbal episodic memory (delayed recall of word list). Node membership to networks marked in gray. Left: Edges positively associated with cognitive performance. Right: Edges negatively associated with cognitive performance. Vis, visual; SM, somatomotor; dAtt, dorsal attention; vAtt, ventral attention; Limb, limbic; Ctr, frontal control; DMN, default mode network

**References to Supplement**

**1.** Morris JC, Heyman A, Mohs RC, et al. The Consortium to Establish a Registry for Alzheimer's Disease (CERAD). Part I. Clinical and neuropsychological assessment of Alzheimer's disease. *Neurology.* 1989; 39(9):1159-1165.

**2.** Tombaugh TN. Trail Making Test A and B: normative data stratified by age and education. *Arch Clin Neuropsychol.* 2004; 19(2):203-214.

**3.** Oswald WD, Fleischmann UM. *Das Nürnberger-Alters-Inventar (NAI)*. Göttingen: Hogrefe; 1997.

**4.** Kalbe E, Kessler J, Calabrese P, et al. DemTect: a new, sensitive cognitive screening test to support the diagnosis of mild cognitive impairment and early dementia. *Int J Geriatr Psychiatry.* 2004; 19(2):136-143.

**5.** Berch DB, Krikorian R, Huha EM. The Corsi block-tapping task: methodological and theoretical considerations. *Brain Cogn.* 1998; 38(3):317-338.
